# *Tropheryma whipplei* in Patients with Pneumonia

**DOI:** 10.3201/eid1602.090610

**Published:** 2010-02

**Authors:** Sabri Bousbia, Laurent Papazian, Jean-Pierre Auffray, Florence Fenollar, Claude Martin, Wenjun Li, Laurent Chiche, Bernard La Scola, Didier Raoult

**Affiliations:** CNRS-IRD Faculté de Médecine, Marseille, France (S. Bousbia, L. Papazian, F. Fenollar, W. Li, B. La Scola, D. Raoult); Hôpitaux Sud, Marseille (L. Papazian, J.P. Auffray, L. Chiche); Hôpital Nord, Marseille (C. Martin)

**Keywords:** Tropheryma whipplei, Whipple disease, Whipple disease diagnosis, ventilator-associated pneumonia, genotyping, bacteria, research

## Abstract

*Tropheryma whipplei* is the etiologic pathogenic agent of Whipple disease (WD), characterized by various clinical signs, such as diarrhea, weight loss, lymphadenopathy, and polyarthritis. PCR-based methods for diagnosis of WD have been developed. *T. whipplei* has been identified in saliva and stool samples from patients with WD and from healthy persons. *T. whipplei* DNA has also been found in bronchoalveolar lavage (BAL) samples of a child with pneumonia. We detected DNA of *T. whipplei* in 6 (3%) of 210 BAL samples collected in intensive care units by using 16S rDNA and specific quantitative PCR. We identified 4 novel genotypes of *T. whipplei*. In 1 case, *T. whipplei* was the only bacterium; in 4 others, it was associated with buccal flora. We suggest that *T. whipplei* should be investigated as an etiologic agent of pneumonia.

## Abstract 2

This bacterium may be an etiologic agent of pneumonia.

*Tropheryma whipplei* is a bacterium widely known to be associated with Whipple disease (WD), which is characterized by various clinical signs such as diarrhea, weight loss, lymphadenopathy, and polyarthritis (*1*). Furthermore, clinical features such as blood culture–negative endocarditis and neurologic lesions have been reported (*2*). Until 1991, WD diagnosis was based essentially on histopathologic observations, characterized by positive periodic acid-Schiff stained inclusions within intestinal macrophages (*3*) and electron microscopy, which has shown the microbiologic etiology of the illness (*4*).

The first molecular identification was made by Wilson et al. in 1991 (the bacterium was uncultured at that time) by using broad-range primers targeting the 16S subunit of rDNA extracted from infected duodenal tissue of a patient with WD (*5*). The bacteria were thus classified within the family *Actinomycetes* (classification was based on 16S rDNA sequence analysis), and the name *Tropheryma whippelii* was proposed (*5*,*6*); the bacterium was later modified to *Tropheryma whipplei* by La Scola et al. (*7*). Investigators then developed several molecular PCR-based methods for the diagnosis of WD. Most of these methods use probes or primer sets targeting parts of the 16S or 23S rDNA genes (*8*).

Successful cultivation of the bacterium by Raoult et al. (*9*) has enabled the adoption of novel diagnostic tools, such as immunohistochemistry and serologic tests (*10*–*12*). Three years after successful cultivation, the genomes of 2 *T. whipplei* strains (T–W08/17, GenBank accession no. NC004551, and Twist, GenBank accession no. NC004572) have been fully sequenced (*13*,*14*). Completion of the sequencing of these 2 genomes has enabled the design of highly specific and sensitive primer pairs that target repeated DNA sequences unique to the *T. whipplei* genome (*15*).

These major advances have enabled the identification of *T. whipplei* DNA in various specimens such as saliva and stools from patients with WD, as well as from asymptomatic carriers, which suggests that the localization of the bacterium is not restricted to the digestive tract and that other organs might be affected (*2*,*16*–*18*). More recently, detection of DNA from *T. whipplei* in bronchoalveolar lavage (BAL) samples from a patient with pneumonia suggests that the bacterium might be involved in respiratory diseases (*19*). We show that *T. whipplei* could be a potential infectious agent for patients admitted to intensive care units (ICUs) and that it can be found in the BAL samples of patients in ICUs.

## Materials and Methods

All case-patients (hereafter patients) were admitted to 1 of 3 ICUs in Marseille, France (1 medical ICU and 2 medicosurgical ICUs) during February 2007 through January 2008. Ages ranged from 18 to 94 years. A total of 210 bronchoalveolar lavage (BAL) fluid samples and 197 blood samples, representing 197 episodes of suspected or confirmed pneumonia, were collected from 134 patients admitted to the 3 ICUs to perform an exhaustive etiologic diagnosis of pneumonia. Bronchoalveolar lavage and blood sampling were collected as previously described (*20*) in the 3 ICUs. The samples were then transported at room temperature to the microbiology laboratory of Timone Hospital, Marseille, France, and preserved at –20°C until handling. Ventilator-associated pneumonia, community-acquired pneumonia, and aspiration pneumonia were defined as previously described (*21*–*23*).

DNA was extracted in a MagnaPure LC workstation (Roche Diagnostics, Meylan, France) by using the MagNa Pure LC DNA Isolation Kit II (Roche Diagnostics). Pellets from BAL fluid samples were mixed with 200 μL of lysis buffer and 50 μL of proteinase K, incubated overnight at 56°C, disrupted for 1 min with glass beads in a MagNa Lyser (Roche Diagnostics), and then processed on the Magnapure LC workstation by the manufacturer’s recommendations. When a BAL sample was cell-poor, to concentrate the bacterial cells, we centrifuged 500 μL of BAL sample for 15 min at maximum speed, discarded 400 μL of the supernatant, then resuspended the pellet in the remaining 100 μL; the extraction procedure was continued as described above.

Standard PCR was performed by using the eubacterial broad-range 16S rDNA primer set 536F: 5′-CAGCAGCCGCGGTAATAC-3′ and rp2: 5′-ACGGCTACCTTGTTACGACTT-3′ (Eurogentec, Seraing, Belgium). PCR was performed in an ABI Themocycler (Applied Biosystems, Courtaboeuf, France).The amplification was done in a 50-μL final volume containing 5 μL extracted DNA, 1X PCR buffer (5 μL), 2 μL of 25 mM MgCl_2_, 200 μM of each dNTP, 0.2 mM of each 536F and rp2 primer, and 1 unit of HotStar Taq DNA Polymerase (QIAGEN, Courtaboeuf, France). Amplification started with an initial incubation at 95°C for 15 min to denature DNA and activate polymerase enzymes, followed by 35 cycles of heating at 95°C for 1 min, annealing at 62°C for 30 s, and extension at 72°C for 90 s. Amplification ended with an extension step at 72°C for 10 min. The PCR products were purified by using the Nucleo-Fast 96 PCR Kit (MACHEREY-NAGEL, Hoerdt, France) as defined by the supplier, and 4 μL of purified PCR product was sequenced in 20 μL final volume containing sequencing buffer, 3.2 pmole of forward (536F) or reverse (rp2) primer, 3 μL of Big Dye Terminator V1.1 mix (Applied Biosystems), and 8 μL of deionized water. Sequencing reactions were purified by using Sephadex Gel-Filtration (Sigma-Aldrich Chimie, Saint Quentin Fallavier, France), and the purified products were sequenced on an ABI PRISM 3130xl genetic analyzer (Applied Biosystems). Sequences obtained were analyzed with Autoassembler software and compared with those available in the GenBank database by using the BLAST program (www.ncbi.nlm.nih.gov/BLAST).

When the BAL fluid was polymicrobial, purified PCR products were cloned into PCR II TA cloning vector (Invitrogen, Cergy Pontoise, France) by using 3 μL of purified PCR products from the previous step as recommended by the manufacturer, and 56–64 white colonies were screened for each specimen. The cloned inserts were amplified with M13 primers set (M13F: 5′-GTAAAACGACGGCCAG-3′, M13R: 5′-CAGGAAACAGCTATGAC-3′) and sequenced as described above.

Quantitative real-time PCR was performed as described by the manufacturer by using a LightCycler instrument (Roche Diagnostics) with the QuantiTect Probe PCR Kit. First, specimens were tested by using the Twhi3F: 5′-TTGTGTATTTGGTATTAGATGAAACAG-3′ and Twhi3R: 5′-CCCTACAATATGAAACAGCCTTTG-3′ primer pair and the specific TaqMan probe Twhi3: 6-FAM-GGGATAGAGCAGGAGGTGTCTGTCTGG-TAMRA. When the specimen was positive in this assay, the result was confirmed by a second quantitative PCR by using the Twhi2F: 5′-TGAGGATGTATCTGTGTATGGGACA-3′ and Twhi2R: 5′-TCCTGTTACAAGCAGTACAAAACAAA-3′ primer set and the Twhi2 probe: 6-FAM-GAGAGATGGGGTGCAGGACAGGG-TAMRA.

Genotyping of *T. whipplei* detected in the BAL fluid samples was conducted as described previously (*24*). Each of the 4 highly variable genomic sequences (HVGSs) obtained from each specimen was compared with those available in the GenBank database and our internal laboratory database to determine their corresponding genotype. The combination of the 4 HVGSs was then analyzed to define the genotype of the bacteria.

Serologic assays were performed by Western blotting. The *T. whipplei* Twist strain was cultivated in axenic medium as previously reported (*25*). Native and deglycosylated samples obtained from the total bacterial extract were prepared for sodium dodecyl sulfate–polyacrylamide gel electrophoresis. The assay was performed to test all immunoglobulins (Ig) total (IgT), including IgG, IgM, and IgA heavy and light chains, as well as to test IgG, IgM, and IgA separately, as previously described (*26*). Detection was performed by using chemiluminescence (ECL Western Blotting Analysis System) and an automated film processor (Hyperprocessor, GE Healthcare, Buckinghamshire, UK). To quantify Western blot bands, we scanned films with an Image Scanner III (GE Healthcare). We performed image analysis by using GelEval 1.21b FrogDance software and ImageJ software (http://rsb.info.nih.gov/ij).

## Results

Bacterial DNA of *T. whipplei* was detected in 6 of 210 BAL fluid samples by standard or quantitative PCR (Table 1). The patients’ ages ranged from 39 to 73 years (mean ± SD 56.83 ± 14.01 years), and all were men (Table 2). One of those 6 specimens (no. 5) was positive for the bacterium in both standard and quantitative PCR assays. For this patient, PCR with broad-range primers showed that *T. whipplei* was the only bacterium identified. Moreover, both quantitative PCR assays showed a high level of the bacterium in this specimen (cycle threshold = 20 with Whi3 probe and cycle threshold = 21 with Whi2 probe; 5.10^5^ copy [Table 1]). This patient was immunocompromised and had community-acquired pneumonia and septic shock (Table 2). He was admitted to the medical ICU for septic shock and acute respiratory distress syndrome complicating community-acquired pneumonia (Figure). He received chemotherapy for difficult-to-treat mediastinal lymphoma. A lobectomy on his upper right lung had been done 1 year before admission for the lymphoma. Lung infiltrates were present when he was admitted to the hospital. The patient was febrile (39.2°C) and hypoxemic (partial pressure of oxygen in arterial blood [PaO_2_] 120 mm Hg for a fraction of inspired oxygen [FiO_2_] at 1), and pancytopenia was evident after initial examination. He received blood products during his ICU stay (9 packed erythrocytes, 4 fresh frozen plasma, 3 platelet transfusions). He was empirically treated by using ticarcillin/clavulanic acid and erythromycin. Hemodynamic status improved rapidly, and administration of vasopressors was stopped by day 2. The patient was finally extubated at day 7 and discharged from the ICU on day 10. He fully recovered after completing a treatment regimen of imipenem, amikacin, and vancomycin.

**Table 1:** PCR test results of bronchoalveolar lavage specimens that were positive for *Tropheryma whipplei* DNA, collected from 6 intensive care unit patients in Marseille, France, February 2007–January 2008*

Specimen no.	16S rDNA for *T. whipplei*	qPCR probe						Other bacteria identified (16S rDNA primer set)	
		Twhi3	Ct	Twhi2	Ct	Load (copy)		Bacterium	Identity, %
5	+	+	20	+	21	5.10^5^		–	99
10	–	+	30	+	31	4,8.10^2^		*Streptococcus pneumoniae*	99
								*Actinobacillus pleuropneumoniae*	99
								*Peptostreptococcus* sp.	99
82	–	+	29	+	28	5,3.10^3^		*Streptococcus* genomosp. C4	99
								Uncultured	99
								*Streptococcus* sp. clone 2.17	89
								*Streptococcus sanguinis*	99
								*Gemella sanguinis*	99
								*Leptotrichia* sp.	95
								*Haemophilus quentini*	99
								Uncultured *Haemophilus* sp.	99
								*Peptostreptococcus* sp.	99
								*Granulicatella para-adiacens*	95
								*Prevotella* sp.	99
								*Prevotella melaninogenica*	99
								*Cloacibacterium normanense*	93
								*Ralstonia solanacearum*	99
								*Haemophilus haemolyticus*	99
								Uncultured *Arcobacter* sp. clone DS126	99
								*Prevotella salivae*	99
								*Streptococcus parasanguis*	99
86	–	+	31	+	29	7,6.10^2^		Uncultured *Porphyromonas* sp. clone 302E06	99
								Uncultured *Capnocytophaga* sp.	95
								*Gemella sanguinis*	99
								*Streptococcus constellatus*	99
								*Prevotella melaninogenica*	91
								Uncultured *Porphyromonas* sp.	93
								*Haemophilus parainfluenzae*	99
								Uncultured *Tannerella* sp.	98
								*Prevotella melaninogenica*	99
								*Granulicatella para-adiacen*s	99
183	–	+	36	+	37	50		*Staphylococcus epidermidis*	99
								*Porphyromonas gingivalis*	99
								*Pseudomonas* sp.	92
								*Eubacterium* sp.	94
								*Streptococcus anginosus*	99
								Uncultured *Neisseria* sp. clone 502G08	99
								*Treponema* sp.	97
								*Clostridium* sp.	97
								*Acidovorax* sp./*Diaphorobacter* sp.	99
								*Eubacterium brachy*	99
								*Gemella haemolysans*	99
								*Mycoplasma orale*	95
								*Comamonas denitrificans*	95
								*Prevotella tannerae*	98
								*Peptostreptococcus micros*	99
209	–	+	35	+	35	70		*Abiotrophia defectiva*	99
								*Acidobacteria* sp.	94
								*Afipia* genosp. 12	97
								*Sphingomonas* sp.	98
								*Pseudomonas stutzeri*	100

*qPCR, quantitative PCR; Ct, cycle threshold.

**Table 2:** Demographic and clinical data on 6 intensive care unit patients with pneumonia from whom bronchoalveolar lavage samples positive for *Tropheryma whipplei* DNA were collected, Marseille, France, February 2007–January 2008*

Specimen no.	Patient age, y/sex	Immunocompromised	Diagnosis	Type of pneumonia	Duration of MV, d	Duration of ICU stay, d	*T. whipplei* genotype	Outcome
5	39/M	Yes (chemotherapy)	Septic shock	CAP	7	10	New	Survived
10	46/M	Yes (splenectomy)	Coma	Aspiration	3	5	3	Survived
82	65/M	No	Coma	Aspiration	16	27	New	Survived
86	74/M	No	Pulmonary embolism	VAP	42	42	New	Died
183	43/M	No	Pancreatitis	VAP	14	16	New	Survived
209	74/M	Yes (corticosteroids)	Spinal cord injury	Aspiration	81	81	ND	Died

*MV, mechanical ventilation; ICU, intensive care unit; CAP, community-acquired pneumonia; VAP, ventilator-associated pneumonia; ND, not determined. †Based on 4 highly variable genomic sequences.

**Figure:**
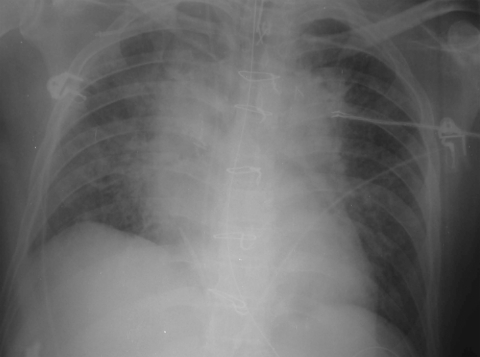
Chest radiograph of patient no. 5, who had community-acquired pneumonia associated with *Tropheryma whipplei*.

Specimens nos. 10, 82, 86, 183, and 209 from 5 other patients were positive for *T. whipplei* in quantitative real-time PCR by using the Twhi3 probe and primer pairs and successfully confirmed by using the Twhi2 probe and primer pairs (Table 1). The bacterial load in each specimen is shown in Table 1. Standard cultures were negative; 10^4^ CFUs were used as the cutoff. The 16S rDNA assay yielded 50 bacteria representing 46 species (Table 1). Of these 46 bacterial species, 15 had <97% 16S rRNA sequence identity with the sequences available in the GenBank database, which suggests a potentially new bacterial species (Table 1). However, despite the high number of the clones screened for these patients, this approach was unable to detect DNA of *T. whipplei*. This result could be explained by the polymicrobial aspect of these specimens caused by the presence of oral bacterial flora, which was proven by the high number of bacteria identified from each specimen (Table 1). Patients 10, 82, 86, and 183 had a typical buccal flora. Two of these 4 patients had aspiration pneumonia; the remaining 2 had ventilator-associated pneumonia. The patient from whom specimen no. 209 was collected had flora of an apparent environment and water origin, but aspiration pneumonia likely developed.

Genotyping of *T. whipplei* was successfully performed for 4 specimens (specimen nos. 5, 10, 82, and 86). Three new *T. whipplei* genotypes were identified in specimen nos. 5, 82, and 86. *T. whipplei* genotype 3 was isolated from specimen 10; this genotype is currently identified in patients with endocarditis caused by *T. whipplei* and in patients with digestive WD (*24*). Another new genotype of *T. whipplei* was identified in specimen no. 183. In this sample, because of the low level of the bacterium, only 2 HVGSs (HVGS1 and HVGS3) were successfully amplified. However, after analysis of the HVGS1 sequence, we concluded that it was a new genotype different from those previously identified. Serologic assays showed no immunoreactivity in the serum of patients whose BAL samples were positive for *T. whipplei* in molecular assays.

## Discussion

We report the use of broad-range primer-based PCR amplifying the 16S rDNA gene followed by cloning and sequencing to identify bacteria associated with pneumonia occurring in mechanically ventilated ICU patients. These results agree with previously published studies (*19*,*27*,*28*) of cystic fibrosis patients, which showed that unusual microbiologic agents can be responsible for pneumonia. The present study shows that these unusual agents of pneumonia can also be identified in mechanically ventilated ICU patients.

Our results show that *T. whipplei* is an etiologic pathogen in pneumonia. We tested samples from all patients positive for *T. whipplei* twice by RTq PCR using different primer pairs and probes. In the broad-range PCR-based assay, samples from the first patient were positive for *T. whipplei* by direct amplification and sequencing of the 16S rDNA gene. Together with the published work of Harris et al., this positive result prompted us to test systematically for *T. whipplei* in all BAL samples collected (*19*). Indeed, as we were conducting our study, Harris et al. reported the detection of *T. whipplei* in the sputum of a child with pneumonia (*19*). Moreover, we were able to genotype *T. whipplei* in 5 specimens (i.e., 4 further amplifications for nos. 5, 10, 82, and 86 and 2 other amplifications for no. 183). Serologic assays showed no immunoreactivity in the serum of the patients of interest. In this age group (61 ± 3.6 years), the percentage of positivity by Western blot is 45% (F. Fenollar, D. Raoult, unpub. data). The absence of immunoreactivity in serum of patients whose BAL fluid was positive for *T. whipplei* in molecular assays shows that *T. whipplei* was probably not present in those patients before the current pneumonia developed. Moreover, antibody response against *T. whipplei* is paradoxal. We recently showed that patients with chronic asymptomatic carriage of *T. whipplei* in fecal specimens had antibodies to *T. whipplei*; patients with WD did not exhibit or exhibited a low reaction to *T. whipplei* (*29*). The lack of antibodies for *T. whipplei* in patients points to a recent infection in nonimmune persons.

The pathogenic role played by *T. whipplei* in pneumonia is difficult to define. In the first patient (specimen no. 5), the role of *T. whipplei* in pneumonia is probable. In fact, it was the only pathogen we detected using the broad-range PCR-based assay, and it was present at a high level in an immunocompromised patient (5.10^5^ copies of the genome) (Table 1). This patient had community-acquired pneumonia when admitted to the hospital. Results for the 5 other case-patients should be interpreted more cautiously. For the patients from whom specimens no. 10, 82, 86, and 183 were collected, amplification of the 16S rDNA gene showed the presence of several other bacteria usually found in oral flora. For the patients from whom specimens 10 and 82 were collected, both of whom had aspiration pneumonia, we hypothesize that *T. whipplei* was part of the oral or digestive flora that contaminated this patient with many other pathogens. It is likely that this was also true for the patients from whom specimens 86 and 183 were collected, both of whom had ventilator-associated pneumonia. For the remaining patient (specimen no. 209), the situation was more complex. In fact, we found oral and dental bacteria, 3 water bacteria, and *Pseudomonas stutzeri*, which is an environmental bacterium. The role of *T. whipplei* in this patient remained unclear.

In summary, we reported a series of patients in whom *T. whipplei* DNA was found in BAL fluids. In 1 patient (specimen no. 5), the role of *T. whipplei* in pneumonia is highly convincing. For the 5 other cases, *T. whipplei* was identified, as were other oral and dental bacteria. The pathogenic role played by *T. whipplei* in the pneumonia for these 5 patients in conjunction with other bacteria is difficult to define but cannot be excluded. Our findings confirm those reported by Harris et al. (*19*), who found *T. whipplei* DNA in the sputum of a child with pneumonia. The role of *T. whipplei* in ICUs remains to be elucidated. *T. whipplei* is an ubiquitous bacterium in the digestive tract, and it has been shown that *T. whipplei* might be present in the saliva of asymptomatic persons (*2*,*30*). It is therefore probable that *T. whipplei* could contribute to the occurrence of aspiration pneumonia along with bacteria present in the oral flora. The presence of *T. whipplei* as the unique identified agent in one of the reported cases is suggestive of a real pathogenicity of this agent.

It is too early to conclude whether *T. whipplei* is an etiologic agent of aspiration or isolated pneumonia. Nevertheless, the existence of *T. whipplei* DNA in ≈3% of BAL fluid samples collected from patients with pneumonia undoubtedly raises questions about its role in the genesis of pneumonia that develops in ICU patients.

## References

[1] Whipple GH. A hitherto undescribed disease characterized anatomically by deposits of fat and fatty acids in the intestinal and mesenteric lymphatic tissues. Bull Johns Hopkins Hosp. 1907;18:382–93.

[2] Fenollar F, Puechal X, Raoult D. Whipple disease. N Engl J Med. 2007;356:55–66. 10.1056/NEJMra062477 17202456

[3] Black-Schaffer B. The tinctoral demonstration of a glycoprotein in Whipple disease. Proc Soc Exp Biol Med. 1949;72:225–7. 15391722 10.3181/00379727-72-17388

[4] Yardley JH, Hendrix TR. Combined electron and light microscopy in Whipple disease. Demonstration of “bacillary bodies” in the intestine. Bull Johns Hopkins Hosp. 1961;109:80–98. 13787237

[5] Wilson KH, Blitchington R, Frothingham R, Wilson JA. Phylogeny of the Whipple’s-disease–associated bacterium. Lancet. 1991;338:474–5. 10.1016/0140-6736(91)90545-Z 1714530

[6] Relman DA, Schmidt TM, MacDermott RP, Falkow S. Identification of the uncultured bacillus of Whipple disease. N Engl J Med. 1992;327:293–301. 1377787 10.1056/nejm199207303270501 PMC13545976

[7] La Scola B, Fenollar F, Fournier PE, Altwegg M, Mallet MN, Raoult D. Description of *Tropheryma whipplei* gen. nov., sp. nov., the Whipple disease bacillus. Int J Syst Evol Microbiol. 2001;51:1471–9. 11491348 10.1099/00207713-51-4-1471

[8] Fenollar F, Raoult D. Whipple disease. Curr Gastroenterol Rep. 2003;5:379–85. 10.1007/s11894-003-0050-6 12959718

[9] Raoult D, Birg ML, La SB, Fournier PE, Enea M, Lepidi H, Cultivation of the bacillus of Whipple disease. N Engl J Med. 2000;342:620–5. 10.1056/NEJM200003023420903 10699161

[10] Raoult D, La SB, Lecocq P, Lepidi H, Fournier PE. Culture and immunological detection of *Tropheryma whippelii* from the duodenum of a patient with Whipple disease. JAMA. 2001;285:1039–43. 10.1001/jama.285.8.1039 11209175

[11] Kowalczewska M, Fenollar F, Lafitte D, Raoult D. Identification of candidate antigen in Whipple disease using a serological proteomic approach. Proteomics. 2006;6:3294–305. 10.1002/pmic.200500171 16637011

[12] Bonhomme CJ, Renesto P, Nandi S, Lynn AM, Raoult D. Serological microarray for a paradoxical diagnostic of Whipple disease. Eur J Clin Microbiol Infect Dis. 2008;27:959–68. 10.1007/s10096-008-0528-0 18594884

[13] Bentley SD, Maiwald M, Murphy LD, Pallen MJ, Yeats CA, Dover LG, Sequencing and analysis of the genome of the Whipple disease bacterium *Tropheryma whipplei.* Lancet. 2003;361:637–44. 10.1016/S0140-6736(03)12597-4 12606174

[14] Raoult D, Ogata H, Audic S, Robert C, Suhre K, Drancourt M, *Tropheryma whipplei* Twist: a human pathogenic actinobacteria with a reduced genome. Genome Res. 2003;13:1800–9. 12902375 10.1101/gr.1474603 PMC403771

[15] Fenollar F, Fournier PE, Robert C, Raoult D. Use of genome selected repeated sequences increases the sensitivity of PCR detection of *Tropheryma whipplei.* J Clin Microbiol. 2004;42:401–3. 10.1128/JCM.42.1.401-403.2004 14715790 PMC321676

[16] Ehrbar HU, Bauerfeind P, Dutly F, Koelz HR, Altwegg M. PCR-positive tests for *Tropheryma whippelii* in patients without Whipple disease. Lancet. 1999;353:2214. 10.1016/S0140-6736(99)01776-6 10392994

[17] Street S, Donoghue HD, Neild GH. *Tropheryma whippelii* DNA in saliva of healthy people. Lancet. 1999;354:1178–9. 10.1016/S0140-6736(99)03065-2 10513716

[18] Dutly F, Hinrikson HP, Seidel T, Morgenegg S, Altwegg M, Bauerfeind P. *Tropheryma whippelii* DNA in saliva of patients without Whipple disease. Infection. 2000;28:219–22. 10.1007/s150100070039 10961527

[19] Harris JK, De Groote MA, Sagel SD, Zemanick ET, Kapsner R, Penvari C, Molecular identification of bacteria in bronchoalveolar lavage fluid from children with cystic fibrosis. Proc Natl Acad Sci U S A. 2007;104:20529–33. 10.1073/pnas.0709804104 18077362 PMC2154465

[20] Berger P, Papazian L, Drancourt M, La SB, Auffray JP, Raoult D. Ameba-associated microorganisms and diagnosis of nosocomial pneumonia. Emerg Infect Dis. 2006;12:248–55. 16494750 10.3201/eid1202.050434 PMC3373093

[21] La Scola B, Boyadjiev I, Greub G, Khamis A, Martin C, Raoult D. Amoeba-resisting bacteria and ventilator-associated pneumonia. Emerg Infect Dis. 2003;9:815–21. 12890321 10.3201/eid0907.030065 PMC3023432

[22] Mandell LA, Wunderink RG, Anzueto A, Bartlett JG, Campbell GD, Dean NC, Infectious Diseases Society of America/American Thoracic Society consensus guidelines on the management of community-acquired pneumonia in adults. Bull Johns Hopkins Hosp. 2007;44(Suppl 2):S27–72. 10.1086/511159 PMC7107997 17278083

[23] Marik PE. Aspiration pneumonitis and aspiration pneumonia. N Engl J Med. 2001;344:665–71. 10.1056/NEJM200103013440908 11228282

[24] Li W, Fenollar F, Rolain JM, Fournier PE, Feurle GE, Mãller C, Genotyping reveals a wide heterogeneity of *Tropheryma whipplei.* Microbiology. 2008;154:521–7. 10.1099/mic.0.2007/011668-0 18227256

[25] Renesto P, Crapoulet N, Ogata H, La SB, Vestris G, Claverie JM, Genome-based design of a cell-free culture medium for *Tropheryma whipplei.* Lancet. 2003;362:447–9. 10.1016/S0140-6736(03)14071-8 12927433

[26] Bonhomme CJ, Renesto P, Desnues B, Ghigo E, Lepidi H, Fourquet P, *Tropheryma whipplei* glycosylation in the pathophysiologic profile of Whipple disease. J Infect Dis. 2009;199:1043–52. 10.1086/597277 19222368

[27] Bahrani-Mougeot FK, Paster BJ, Coleman S, Barbuto S, Brennan MT, Noll J, Molecular analysis of oral and respiratory bacterial species associated with ventilator-associated pneumonia. J Clin Microbiol. 2007;45:1588–93. 10.1128/JCM.01963-06 17301280 PMC1865865

[28] Bittar F, Richet H, Dubus JC, Reynaud-Gaubert M, Stremler N, Sarles J, Molecular detection of multiple emerging pathogens in sputa from cystic fibrosis patients. PLoS One. 2008;3:e2908. 10.1371/journal.pone.0002908 18682840 PMC2483419

[29] Fenollar F, Amphoux B, Raoult D. A paradoxical *Tropheryma whipplei* Western blot differentiates patients with Whipple disease from asymptomatic carriers. Clin Infect Dis. 2009;49:717–23. 10.1086/604717 19635029

[30] Schneider T, Moos V, Loddenkemper C, Marth T, Fenollar F, Raoult D. Whipple disease: new aspects of pathogenesis and treatment. Lancet Infect Dis. 2008;8:179–90. 10.1016/S1473-3099(08)70042-2 18291339

